# Development and psychometric testing of an abridged version of Dundee Ready Educational Environment Measure (DREEM)

**DOI:** 10.1186/s12199-018-0702-7

**Published:** 2018-04-17

**Authors:** Kathiresan Jeyashree, Hemant Deepak Shewade, Soundappan Kathirvel

**Affiliations:** 1 Department of Community Medicine, Velammal Medical College Hospital and Research Institute, Madurai, 625009 India; 2 Department of Operational Research, International Union Against Tuberculosis and Lung Disease (The Union), South-East Asia Office, C-6 Qutub Institutional Area, New Delhi, 110016 India; 3 Department of Community Medicine, School of Public Health, Postgraduate Institute of Medical Education and Research (PGIMER), Chandigarh UT, India

**Keywords:** DREEM, Abridged version, Psychometric testing, Confirmatory factor analysis, Educational environment

## Abstract

**Background:**

Dundee Ready Educational Environment Measure (DREEM) is a 50-item tool to assess the educational environment of medical institutions as perceived by the students. This cross-sectional study developed and validated an abridged version of the DREEM-50 with an aim to have a less resource-intensive (time, manpower), yet valid and reliable, version of DREEM-50 while also avoiding respondent fatigue.

**Methods:**

A methodology similar to that used in the development of WHO-BREF was adopted to develop the abridged version of DREEM. Medical students (*n* = 418) from a private teaching hospital in Madurai, India, were divided into two groups. Group I (*n* = 277) participated in the development of the abridged version. This was performed by domain-wise selection of items that had the highest item-total correlation. Group II (*n* = 141) participated in the testing of the abridged version for construct validity, internal consistency and test-retest reliability. Confirmatory factor analysis was performed to assess the construct validity of DREEM-12.

**Results:**

The abridged version had 12 items (DREEM-12) spread over all five domains in DREEM-50. DREEM-12 explained 77.4% of the variance in DREEM-50 scores. Correlation between total scores of DREEM-50 and DREEM-12 was 0.88 (*p* < 0.001). Confirmatory factor analysis of DREEM-12 construct was statistically significant (LR test of model vs. saturated *p* = 0.0006). The internal consistency of DREEM-12 was 0.83. The test-retest reliability of DREEM-12 was 0.595, *p* < 0.001.

**Conclusion:**

DREEM-12 is a valid and reliable tool for use in educational research. Future research using DREEM-12 will establish its validity and reliability across different settings.

## Background

Education environment refers to the diverse physical locations in which the students learn. The educational environment of an institution has direct and indirect influence(s) upon the knowledge gained by the students including the motivation to learn, personal safety and their well-being [1]. Dundee Ready Educational Environment Measure (DREEM) is one of the tools developed specifically to assess the educational environment of medical institutions as perceived by the students.

DREEM has 50 items grouped under five domains namely students’ perception of learning (SPoL; *n* = 12), students’ perception of teachers (SpoT; *n* = 11), students’ academic self-perception (SASP; *n* = 8), students’ perception of atmosphere (SPoA; *n* = 12), and students’ social self-perception (SSP; *n* = 7). It is a generic and culturally non-specific tool which has been translated and validated in at least eight languages and is used worldwide [2]. In addition to being used to diagnose the deficiencies of the current educational environment, DREEM has also been used to compare different groups, to monitor the same cohort over a period of time and to assess factors influencing the educational environment [3, 4]. DREEM was used among undergraduates [5] including nursing [6, 7] and dental students and various post graduate medical students [8, 9] to explore their perceptions of educational environment.

Though universally accepted and used, investigators followed their own analysis and reporting system of DREEM data [10]. Few studies questioned the five-factor structure and construct validity of the original DREEM tool and also tried to develop abridged versions of DREEM [11–14]. The other studies have developed an abridged version but the properties of the abridged version have not been tested on another cohort. These abridged versions have not been validated nor have they been widely in use.

Our study developed and validated an abridged version of the DREEM-50 with an aim to have a less resource-intensive (time, manpower), yet valid and reliable, version of DREEM-50 while also avoiding respondent fatigue.

## Methods

### Study design and setting

A cross-sectional study was conducted for development of an abridged version of DREEM at a private, tertiary care teaching institute in Madurai, a city in Tamil Nadu state of India. It is one of the two major and the only private institution teaching medicine and allied health sciences in the city. Every year, 150 students are selected through a competitive examination for the undergraduate course in medicine—Bachelor of Medicine and Bachelor of Surgery (M.B, B.S.).

### Study participants

A list of the 450 undergraduate medical students currently enrolled in the institute (third, fourth and sixth semester) as on August 2016 was obtained, and all students were invited to participate in the study. These students were divided into two groups group I (students of the fourth and seventh semester, *n* = 300) and group II (students of third semester, *n* = 150).

### Data collection

The DREEM-50 item version was administered to both groups after obtaining written informed consent, and two separate datasets were maintained. The author was present during the consent process to ensure that clarifications were provided to queries, if any. Students who did not consent and those unavailable after three attempts of contact were excluded from the study.

### Statistical analysis

Double data entry and validation was done using EpiData version 3.1 (EpiData Association, Odense, Denmark). Items that had to be reverse coded were done so before entry. The data was analysed using STATA (version 12.1, copyright 1985–2011 StataCorp LP USA, serial number: 30120504773). Number and proportion were calculated for sociodemographic characteristics like gender and semester. Mean and standard deviation were calculated for age and for score of each item, domain and total scale of DREEM-full and abridged version.

#### Development of an abridged version

For the development of the abridged version, the authors followed a procedure similar to that adopted in the development of WHOQOL-BREF, an abridged version of WHOQOL-100, which measures quality of life (QOL) [15]. Item-total correlation (r) and domain-total correlation (r) were calculated (group I dataset). Two items with the highest item-total correlation values were selected from each of the five domains of the DREEM questionnaire. When two items had the same correlation value, both were included. Thus, the authors arrived at a 12-item version of DREEM (DREEM-12). The face and content validity of this abridged version was assessed by the three authors, in their capacity as subject experts each with a minimum of 3 years’ experience in medical education.

#### Validity and reliability of DREEM-12

DREEM-50 and DREEM-12 scores were calculated on group II dataset. Internal consistency was measured using Cronbach’s alpha. The percentage of variance in DREEM-50 that is explained by the abridged version was calculated using R square. DREEM-12 alone was administered on group II to get the second set of scores of DREEM-12 to check for test-retest reliability (*r*).

Confirmatory factor analysis was performed to assess the construct validity of DREEM-12. A five-factor first-order model was fit to assess the DREEM-12 structure using the items selected for each DREEM-12 component (items), i.e. SPoT (two), SPoT (three), SASP (three), SPoE (two) and SSP (two).

### Ethics

Ethics approval was obtained from institute ethics committee of Velammal medical college hospital and research institute, Madurai, Tamil Nadu. Written informed consent was taken from the students and the consent process was approved by the ethics committee.

## Results

A total of 277 students from group I participated in the development of the abridged version and 141 students from group II in the validation and reliability of the abridged version. The age and gender distribution of the study population is given in Table 1.

**Table 1:** Profile of study participants - undergraduate medical students from Madurai, India in 2016 (*n* = 418)

Characteristics		Group I *n* = 277	Group II *n* = 141
Mean age (SD) in years		19.2 (0.8)	18.1 (0.64)
Gender (%)	Male	104 (37.5)	54 (38.3)
	Female	173 (62.5)	87 (61.7)

Group I–fourth and sixth semester students; group II–third semester students

*SD* standard deviation

The item-total correlation and domain-total correlation of the items selected for inclusion in DREEM-12 are given in Table 2. Correlation between total scores of DREEM-50 and DREEM-12 was 0.88 (*p* < 0.001). Domain–domain correlation between DREEM-50 and DREEM-12 were 0.75 (SPoL), 0.7 (SPoT), 0.79 (SASP), 0.66 (SPoA) and 0.62 (SSP). Regression analysis revealed that DREEM-12 explained 77.4% of the variance in DREEM-50. The internal consistency of DREEM-12 was 0.83 as compared to 0.86 of DREEM-50.

**Table 2:** Domain-wise list of items in DREEM-50 that had the highest item-total and domain-total correlations

Domain	Item no	Item	Item-total correlation	Domain-total correlation
Students’ perception of learning	44	The teaching encourages me to be an active learner	0.646	0.711
	22	The teaching helps to develop my confidence	0.556	
Students’ perception of teachers	2	The course organisers are knowledgeable	0.510	0.611
	37	The course organisers give clear examples	0.450	
	18	The course organisers have good communication skills with student	0.450	
Students’ academic self-perception	21	I feel I am being well prepared for my profession	0.435	0.599
	41	My problem solving skills are being well developed here	0.430	
	45	Much of what I have to learn seems relevant to a career in healthcare	0.430	
Students’ perception of atmosphere	36	I am able to concentrate well	0.500	0.633
	43	The atmosphere motivates me as a learner	0.487	
Students’ social self-perception	3	There is a good support system for students who get stressed	0.419	0.556
	19	My social life is good	0.416	

The item wise and overall scoring system of DREEM-12 was devised in line with the DREEM-50 (Table 3). When calculated for group II, DREEM-50 and DREEM-12 scores yielded the same interpretations on the students’ perceptions about the educational environment: overall and at domain level (Table 4).The test-retest reliability of DREEM-12 was 0.595, *p* < 0.001.

**Table 3:** Items selected to form DREEM-12 and the applicable scoring system

Domain (Maximum score)	Item No.	Item	Scoring
Student’s Perception of Learning (8)	22 44	The teaching helps to develop my confidence The teaching encourages me to be an active learner	0–2 Very Poor 3–4 Teaching is viewed negatively 5–6 A more positive perception 7–8 Teaching highly thought of
Student’s Perception of Teachers (12)	2 18 37	The course organizers are knowledgeable The course organizers have good communication skills with student The course organizers give clear examples	0–2 Abysmal 4–6 In need of some retraining 7–9 Moving in the right direction 10–12 Model course organizers
Students’ Academic Self-Perception (12)	21 41 45	I feel I am being well prepared for my profession My problem solving skills are being well developed here Much of what I have to learn seems relevant to a career in healthcare	0–3 Feelings of total failure 4–6 Many negative aspects 7–9 Feeling more on the positive side 10–12 Confident
Students’ Perceptions of Atmosphere (8)	36 43	I am able to concentrate well The atmosphere motivates me as a learner	0–2 A terrible environment 3–4 There are many issues which need changing 5–6 A more positive attitude 7–8 A good feeling overall
Students’ Social Self Perceptions (8)	3 19	There is a good support system for students who get stressed My social life is good	0–2 Miserable 3–4 Not a nice place 5–6 Not too bad 7–8 Very good socially
Overall scoring for interpretation of educational environment: 0–12 Very Poor 13–24 Plenty of Problems 25–36 More Positive than Negative 37–48 Excellent

**Table 4:** Comparison of DREEM-50 and DREEM-12 scores and their interpretation calculated for group II

SN	Domain	DREEM-50		DREEM-12	
		Mean score (SD)	Interpretation	Mean score (SD)	Interpretation
1	Students’ perception of learning	30.5 (5.4)	A more positive perception	5.1 (1.6)	A more positive perception
2	Students’ perception of teachers	25 (4.8)	Moving in the right direction	8.4 (2.1)	Moving in the right direction
3	Students’ academic self-perception	21.1 (4.2)	Feeling more on the positive side	8.1 (1.9)	Feeling more on the positive side
4	Students’ perception of atmosphere	28.2 (5.9)	A more positive attitude	4.8 (1.6)	A more positive attitude
5	Students’ social self-perception	16.8 (3.6)	Not too bad	4.7 (1.7)	Not too bad
Total		121.7 (18)	More Positive than Negative	31.1 (6.3)	More Positive than Negative

*SD* standard deviation

**Fig. 1:**
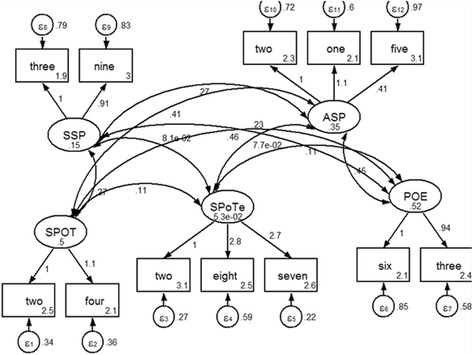
Confirmatory factor analysis of DREEM-12 construct. SSP-Social Self Perception, ASP-Academic Self perception, SPOT-Students’ Perception of Teachers, SPOTe-Students’ Perception of Teaching, POE- Perception of Environment three-3, ninet-19, twone-21, fone-41, ffive-45, twtwo-22, ffour-44, two-2, eightn-18, tseven-37, tsix-36, fthree-43. LR test of model vs. saturated: chi2(44)=80.88, Prob > chi2 = 0.0006

Confirmatory factor analysis of DREEM-12 construct was statistically significant (LR test of model vs. saturated: *χ*^2^(44) = 80.88, Prob> *χ*^2^ = 0.0006) (Fig. 1).

## Discussion

This study developed and validated DREEM-12, an abridged version of DREEM-50, using two subsets of a population of undergraduate medical students and established good model fit of DREEM-12 by confirmatory factor analysis.

Our study, besides developing an abridged version of DREEM-50, has also confirmed its factor structure and established its validity and reliability. We have employed robust data quality management, through double data entry and validation, and statistical techniques to prove the same. Our study has enrolled students from different years of medical education and thus can be considered representative of the perceptions of students in different stages of medical education.

The study was limited to only students from a single teaching institution in India. However, ours being a study on educational environment, it is also necessary to point out that the quality standards of a medical college with respect to curriculum, teaching methodology, infrastructure, staffing and assessment methods are strictly governed to be uniform by the Medical Council of India. These standards are uniform for all the colleges countrywide. All colleges, government and private, are to abide by these standards, failing which the recognition of the institute is cancelled and the given institute will no more be able to admit candidates. Hence, it will be safe to say that the educational environment offered in colleges across the country are to be uniform. The students are admitted into the undergraduate medical course by a nationwide common competitive entrance examination—National eligibility entrance test. The students do come from different sociodemographic and cultural backgrounds in different parts of the country. But, we do not expect that to significantly affect their perception of their environment. Thus, while the internal validity of our study is commendable, further multi-centric research is required to establish the generalisability of the results outside the country.

The psychometric properties of original DREEM-50 were investigated in few studies. Confirmatory factor analysis found that the five-factor structure of DREEM-50 showed poor model fit in studies conducted in Malaysia and Australia [12, 14]. Both these studies came up with an abridged version of DREEM-50 with a good model fit (Table 5). The Malaysian study derived a 17-item abridged version with good model fit with each domain having three items except the students’ perception of atmosphere which had five items. Similarly, the Australian study derived 12-item abridged version with only four domains after removing student’s social self-perception. The difference in the items chosen in our study, Malaysian and Australian abridged versions might have been due to the following reasons- the methodology of choosing items into the abridged version was different in the three studies. In our study, we had followed the method used in development of WHO QOL BREF. We included items based on their item-total correlations. While the Malaysian version retained the five-domain structure of the original DREEM, the Australian version had done away with the social self-perception domain. Since our study revealed equally good domain-total correlation for all domains, similar to the Malaysian study, we chose to retain all domains in our abridged version. Eight (items 3, 19, 22, 37, 41, 43, 44 and 45) out of the 12 items selected in our study (Table 3) also figure in the Malaysian 17-item version [11, 12]. None of the items in the Australian abridged version are present in the current DREEM-12 except for item 22 [14]. Our abridged version is short like the Australian version and maintained the five-domain structure of the original version like the Malaysian version.

**Table 5:** Comparison of items in the abridged version of DREEM developed in our study with those in Malaysian and Australian versions

Domains	Item number(s)		
	India (current study)	Malaysia	Australia
Students’ perception of learning	22, 44	20, 22, 44	13, 22, 24, 48
Students’ perception of teachers	2, 18, 37	6, 37, 40	8, 29, 32, 39
Students’ academic self-perception	21, 41, 45	26, 41, 45	21
Students’ perception of atmosphere	36, 43	30, 33, 42, 43, 49	30, 34, 35
Students’ social self-perception	3, 19	3, 19, 46	–
Total items	12	17	12

A study from Ireland also mentioned the poor construct validity of the DREEM-50 and found some 17 items having poor fit indices (< 0.7). This study failed to propose an abridged version (33 items) mentioning two reasons, namely, the weakest items may not be same for different settings and fear of affecting the factor structure [13].

Reports on test-retest reliability of DREEM are variable. A study from Brazil reported moderate test-retest reliability of 0.43 [16]. Contrarily, a study validating the Greek translation of DREEM, reported 0.90 [17]. While a generally accepted cut-off of 0.7 is followed, there are no standards laid for test-retest reliability cut off for instruments measuring psychological parameters [18].

## Conclusion

DREEM-12, developed as an abridged version of DREEM-50, was found to explain 77.5% of the variance of the scores of the original version and also retained its factor structure, validity and reliability. Future research using DREEM-12 will establish its validity and reliability across different settings.

## Abbreviations

DREEM: Dundee Ready Educational Environment Measure
M.B., B.S.: Bachelor of Medicine and Bachelor of Surgery
QOL: Quality of life
SASP: Students’ academic self-perception
SPoA: Students’ perception of atmosphere
SPoL: Students’ perception of learning
SPoT: Students’ perception of teachers
SSP: Students’ social self-perception
WHOQOL-BREF: World Health organisation Quality of Life- BREF
